# Magnetic resonance elastography of the human brain using a multiphase DENSE acquisition

**DOI:** 10.1002/mrm.27672

**Published:** 2019-01-29

**Authors:** Johannes Strasser, Michaela Tanja Haindl, Rudolf Stollberger, Franz Fazekas, Stefan Ropele

**Affiliations:** ^1^ Department of Neurology Medical University of Graz Graz Austria; ^2^ Institute of Medical Engineering Graz University of Technology Graz Austria

**Keywords:** brain, low‐frequency MR elastography, multiphase DENSE‐MRE, shear waves

## Abstract

**Purpose:**

In magnetic resonance elastography (MRE), a series of time‐shifted images is acquired at specific phase offsets in relation to an induced mechanical excitation. To efficiently gather the set of phase offset images and to overcome limitations due to prolonged TEs and related susceptibility artifacts at low‐frequency MRE, we developed an improved displacement encoding with a stimulated echoes (DENSE) method.

**Methods:**

The proposed multiphase DENSE‐MRE acquisition scheme allows full sampling of the wave propagation in 1 encoding direction during each TR using multiple readouts at specific phase offsets. With this approach, all phase offsets can be imaged in 1 TR without the need for whole sequence repetitions at time‐shifted offsets relative to the excitation motion. We tested this technique in phantom experiments with 60 Hz and in the brain of 4 volunteers using 20‐Hz harmonic excitation.

**Results:**

Three‐dimensional wave propagation could be acquired in 7 minutes 30 seconds. Following background phase elimination, clear wave images were obtained, showing the propagation of the waves over time. Calculated shear modulus maps of the phantom matched well to the maps obtained by conventional gradient‐echo MRE. In the brain, low‐frequency DENSE‐MRE images were free of susceptibility‐induced artifacts and the calculated maps showed a median global complex shear modulus magnitude of 0.72 kPa and phase angle of 1.03 rad across volunteers.

**Conclusion:**

The proposed multiphase DENSE approach allows efficient low‐frequency MRE with short TEs and is well‐suited for low‐frequency MRE of the human brain.

## 1 INTRODUCTION

Magnetic resonance elastography (MRE) aims at assessing mechanical properties of tissues in terms of the complex shear modulus by analyzing the propagation of externally induced mechanical waves.^1^, ^2^, ^3^, ^4^, ^5^, ^6^ It has been demonstrated that the mechanical properties of tissue vary with different pathologies, which makes MRE an interesting candidate for assessing disease‐related tissue changes.^5^, ^6^ Although MRE was first used preferentially for exploring the liver, its application has subsequently extended to other organs including the human brain.^5^, ^6^ Recent MRE studies investigated the mechanical properties of the normal brain, but also searched for tissue changes caused by various neurological diseases including multiple sclerosis, Alzheimer’s disease, and glioblastoma.^6^, ^7^, ^8^

In cerebral MRE, an external actuator is used to transmit vibrations to the brain with applied driving frequencies typically in the range of 10 Hz to 100 Hz, but primarily around 50 Hz to 60 Hz.^9^ Following continuous harmonic excitation, the pattern of the resulting shear waves can be captured through motion‐encoding MRI sequences, with the tissue motion information being usually encoded in the MR phase images.^5^, ^6^, ^9^ Typical acquisition schemes for such an endeavor include gradient‐echo (GRE) or spin‐echo sequences—also with readout strategies like EPI—adapted with additional bipolar motion‐encoding gradients and synchronization to the external vibration.^6^, ^9^ In a conventional setting, the oscillation frequency of the motion‐encoding gradients is directly related to the frequency of the mechanical excitation.^4^ Recently, there is an emerging trend toward lower excitation frequencies (< 25 Hz), because low‐frequency MRE could overcome shear wave attenuation issues in deeper regions of the brain.^10^ This allows the shear waves to travel deeper into the brain tissue due to less damping,^10^ whereby larger areas of the brain cross section can be used for shear modulus calculations. In addition, low excitation frequencies are beneficial in brain MRE, as the wavelengths of the propagating mechanical waves become shortened in softer tissues and brain tissues stiffness is reduced in several neurological diseases such as multiple sclerosis, Parkinson’s disease, and Alzheimer’s disease.^10^

When assessing lower vibration frequencies with conventional motion‐encoding MRE sequences, however, this results in longer TEs and therefore in a signal loss due to T_2_ and T_2_^*^ decay and related susceptibility artifacts. To achieve TEs shorter than the vibration wave period, fractional encoding of the harmonic motion was proposed, in which the motion‐encoding gradient duration is decoupled from the wave period.^10^, ^11^, ^12^ However, instead of conventional motion encoding, we propose to depict the propagation of the shear waves with displacement encoding using a stimulated echo (DENSE),^13^, ^14^ which allows one to assess the extent of tissue displacement between 2 points in time. A significant advantage of this approach is that the encoding gradients have a very short duration. Furthermore, as in fractional encoding,^12^ the TE and the encoding gradients in DENSE‐MRE are independent of the vibration wave period.^14^

The application of stimulated‐echo MRE was already proposed by Plewes et al for breast cancer investigations^15^ and by Robert et al for DENSE‐MRE of the human heart.^14^ However, to obtain the time‐resolved propagation of the vibration‐induced waves in MRE, a set of time‐shifted snapshots (phase offsets) of the waves has to be acquired.^5^, ^6^, ^14^ This can result in a considerable increase of the total acquisition time, depending on the number of gathered phase‐offset images. We present an accelerated approach that uses multiple displacement‐encoded readouts following a single DENSE preparation during each TR. With the consecutive readouts, the vibration can be sampled efficiently at different points in time without the need of whole‐sequence repetitions for gathering the phase offsets.

In this paper, we provide a detailed description of the multiphase offset readout concept in DENSE‐MRE as an efficient acquisition scheme for multislice MRE. We test it in phantom experiments and compare the obtained results with those generated using a conventional GRE‐MRE sequence. Furthermore, we demonstrate the applicability of the multiphase DENSE‐MRE approach to brain MRE first with results from investigations in healthy volunteers.

## 2 METHODS

### 2.1 Multiphase DENSE‐MRE sequence

The concept of stimulated‐echo MRE and DENSE‐MRE has been well described.^14^, ^15^ The acquisition scheme can be summarized as follows. In a preparation part consisting of 2 RF pulses with a gradient (G1) in between, the tissue’s current position is encoded in the longitudinal magnetization. Following a mixing time (TM), the prepared longitudinal magnetization can be read out with another RF pulse and a second gradient (G2) that corresponds to G1 with respect to strength and duration. The motion encoding is provided by G1 and G2. Any tissue displacement along the direction of these gradients that occurred between G1 and G2 leads to a corresponding phase shift in the resulting phase image. The sensitivity for spatial displacement encoding and therefore the relative phase shift is related directly to the applied gradient moment.

**Figure 1:**
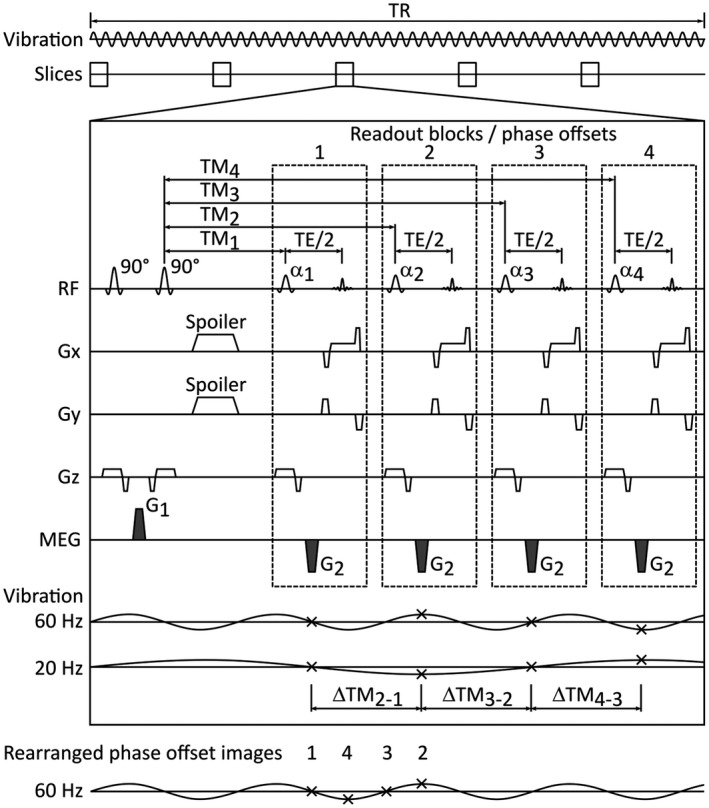
Schematic illustration of 1 TR of the multiphase displacement encoding with a stimulated echo magnetic resonance elastography (DENSE‐MRE) sequence exemplarily for 4 phase‐offset images and 5 slices. Continuous vibration is applied, lasting over all repeated TRs. In each of the interleaved multislice acquisitions, motion information is gathered for all phase offsets. The longitudinal magnetization is prepared by the first 2 90° RF pulses and the motion‐encoding gradient (MEG) G1 in between. At each mixing time (TM), a flip angle α_i_ flips the prepared magnetization back onto the transverse plane, and together with a second gradient G2, the tissue’s displacement is encoded in the respective phase image. This arrangement allows the acquisition of the time‐resolved wave propagation information in a single TR interval. The vibration wave sampling points (marked as crosses) are thereby defined using the differences in TM (∆TM). A rearrangement of the phase offsets can be performed after the acquisition to achieve ascending sampling point ordering. Remaining TR time can be filled with interleaved multislice acquisitions

To sample the mechanical waves in MRE, a set of images is acquired that shows the wave propagation at several equidistant points in time over a complete wave period.^4^ These images are commonly called phase offset images, as each image has a distinct phase offset in relation to the excitation wave.^5^ In conventional MRE and DENSE‐MRE, such a set of phase offset images is collected with repeated acquisitions and different delays between vibration and motion encoding.^5^, ^6^, ^14^ Thus, each phase offset shows a different snapshot of the propagating wave. To improve efficiency, in the multiphase DENSE‐MRE this set of phase offset images can be acquired without the need of repeated scans but in a single sequence run. In DENSE‐MRE, after preparation and mixing time, instead of applying just a single readout with a 90° flip angle RF pulse, a series of readout blocks with lower flip angle RF pulses can be executed during each TR. This is illustrated in the sequence diagram in Figure 1. Each of these readout blocks includes a gradient G2 and is acquired after a distinct TM. The motion encodings in the image series thereby result from the gradient G1 in the preparation part, together with the respective gradients G2 in the series of readout blocks. The time points of the gradients G2 define the time‐resolved sampling of the motion in this process and have to be related to the vibration. In contrast, the time point of G1 does not have to fit a distinct point on the wave motion timeline. With this multiphase approach, all of the consecutive readout blocks have the same effective TE while probing the wave at different phase offsets. The phase offsets between the sampling points of the excitation motion are therefore not achieved by repeated measurements with variable trigger delays, but by an appropriate set of TMs within each TR of a single sequence execution. Although the signal intensities of the succeeding readouts are affected differently by T_1_ relaxation, which influences the magnitude images only, the displacement information is not subject to the T_1_ relaxation because it is encoded in the phase images. However, because the noise in the phase images is related to the magnitude images, a variable flip angle scheme^16^ can be applied to obtain a constant signal intensity. To acquire a set of phase offset images with equal sampling point distribution over the entire vibration period, the differences between the TMs (∆TM) have to be matched accordingly. The sampling point ordering thereby does not have to be strictly ascending during acquisition but may vary depending on the used excitation frequency and the duration of a readout block. As a consequence, reordering of the image series may be necessary during postprocessing.

### 2.2 Phantom measurements

The multiphase DENSE‐MRE sequence was implemented and tested on a 3T whole‐body MR scanner (Siemens Prisma fit; Siemens Healthcare, Erlangen, Germany) using a 20‐channel head coil.

Mechanical excitation was achieved using a high‐power wave generator (CGC Instruments, Chemnitz, Germany) and a piezoelectric actuator (SPL Spindel und Praezisionslager GmbH, Ebersbach, Germany). The actuator was placed on the feet end of the MR scanner’s patient table. Using a rigid rod, the vibration was transmitted to a head rocker. The phantom was placed within the rocker and fixated to achieve good mechanical coupling. A continuous sinusoidal vibration with a frequency of 60 Hz was applied throughout the sequence execution. To guarantee steady state and synchronous vibration conditions during the whole acquisition, the multiphase DENSE‐MRE sequence was synchronized with the control unit for the actuator. The actuator was triggered by the MR sequence at integer multiples of the applied excitation wave period.

The multiphase DENSE sequence was tested on an elastic phantom constructed inside a stiff plastic box. The elastic material consisted of agar (Electran Agarose DNA Grade, VWR International BVBA, Leuven, Belgium) in different agar/water concentrations. Two stiff inclusions with 0.6% agar (0.6 g agar stirred in 100 mL water) were placed inside a soft‐surrounding 0.3% agar gel matrix. Various geometrical shapes were defined for the 2 inclusions with individual lengths. The rectangular inclusion occupied almost the whole length of the phantom, whereas the cylindrical inclusion ranged over just a part of the phantom, to investigate also partial‐volume effects in the multislice acquisition.

Five transversal slices and 4 phase offsets per slice were acquired using the proposed acquisition scheme. Together with a TR of 2500 ms and interleaved multislice image acquisition, this allowed capturing the same sampling points in time of the wave field in all slices.

Displacement information was gathered using trapezoidal shaped motion‐encoding gradients with 34‐mT/m gradient amplitude and 0.7‐ms duration. This led to a motion‐encoding sensitivity of 0.1 mm/rad. Gradient polarity was switched between G1 and G2 as proposed in Ref 14. Following the preparation part, spoiler gradients were set on the readout and phase‐encoding axis to ensure adequate spoiling of the residual transverse magnetization, to avoid an unwanted spin‐echo formation. Because these spoiler gradients do not affect the prepared longitudinal magnetization, they do not contribute to the motion encoding. The wave propagation has been sampled at 4 equidistant points in time, by setting the 4 mixing times TM_i_ to 8 ms, 20.5 ms, 33 ms, and 45.5 ms (Table 1). After every mixing time, a readout block started with a TE part TE/2 of 3.8 ms, and 600 Hz/Px bandwidth. Flip angles α_i_ were set to 30°, 35°, 45°, and 90° for each readout block, respectively, which provided a constant signal intensity for a T1 of 800 ms. The experiments were performed with 300 × 300 mm FOV and a matrix size of 128 × 128, resulting in a spatial resolution of 2.3 × 2.3 mm in plane with 4‐mm slice thickness. For all investigations we used a partial Fourier factor of 6/8 and parallel imaging GRAPPA factor 2. The sequence was executed 3 times with alteration of the motion‐encoding gradient axis to sample 3D wave propagation. The acquisition time for 1 encoding direction was 2 minutes 30 seconds, resulting in a total acquisition time of 7 minutes 30 seconds.

**Table 1:** Reordering schemes of the phase‐offset images for the phantom and in vivo measurements

TM_i_ (ms)	Difference Between TM_i_ and TM_1_ (ms)	Sampling Ordering per Excitation Frequency	
		60 Hz (Phantom)	20 Hz (In Vivo)
8	0	1	1
20.5	12.5	4	2
33	25	3	3
45.5	37.5	2	4

In addition to the proposed multiphase DENSE‐MRE approach, the imaging protocol consisted of a localizer and a T_2_‐weighted fast spin‐echo sequence with identical geometric imaging parameters. Additionally, comparative measurements for 5 slices were performed with a conventional GRE‐MRE sequence. The GRE‐MRE sequence enabled motion encoding in *z*‐direction and 4 trigger‐shifted phase offsets, each acquired twice with inverted motion‐encoding gradient polarity, resulting in 4 phase‐difference wave images. Related imaging parameters were a TE of 22.8 ms, a TR of 50 ms, and the same geometric settings as for the multiphase DENSE‐MRE. The total acquisition time for the GRE‐MRE was 2 minutes 12 seconds for the 5 slices.

### 2.3 Image processing

The following processing steps were performed for each slice and motion‐encoding direction of the multiphase DENSE‐MRE images. First, the raw phase images were converted from DICOM integers to radians. Then, a rearrangement of the data set along the time axis was performed to achieve a temporally ascending wave sampling order, as outlined in Table 1. For visualization of the wave propagation, constant background phase was removed from all of the phase offsets by mean value subtraction. Spatial mean values were subtracted in every image and the temporal mean values were removed along the time axis of the phase‐offset series to eliminate the background phase. All subtractions were performed in the complex domain as arctangent (exp(i*φ)*exp(−i*φ_mean_)); thus, it additionally removed the few phase wraps of the raw phase images and no further phase unwrapping of the wave images was needed. To extract the frequency component corresponding to the excitation frequency, discrete Fourier transform was performed from time to frequency domain along the wave‐sampling point axis. A complex wave image was extracted as first harmonic of the Fourier transformed data at the excitation frequency component. The MRE processing was performed using a 2D multifrequency dual elasto visco (MDEV) inversion algorithm,^7^ executed with the applied single‐excitation frequency and all displacement‐encoding directions. A constant density of the material of 1 g/cm^3^ was assumed for the shear modulus estimation. During inversion, the images were smoothed using a 2D Gaussian filter (5 × 5 pixel kernel, sigma of 1 pixel).^10^ Further on, the MDEV algorithm contained gradient‐based unwrapping and a 2D Butterworth low‐pass filter with a threshold of 100‐m^−1^ suppressed higher wavelengths within the processed images as described in Ref 7. As a result of the inversion, the complex shear modulus G* was computed in terms of its magnitude |G*| (representing the material firmness) and phase angle φ (defining the viscous material properties).^7^ The shear modulus maps of the phantom were then smoothed using a 2D median filter with a 5 × 5 pixel kernel. Regions of interest (ROIs) were drawn manually on each slice of the T_2_‐weighted images for the higher agar concentrated inclusions as well as in the lower agar concentrated surrounding area. The resulting ROIs of all slices were then combined (for the inclusions and surrounding area separately). The ROIs were copied to the |G*| and φ maps to assess the mean values for the agar phantom and its inclusions. To compare the G* maps from the multiphase DENSE‐MRE to the maps from the GRE‐MRE, MDEV inversion was additionally performed using just the *z*‐motion encoded data set. The GRE‐MRE phase offset images were processed to G* maps using the same MDEV inversion and filtering as described for the multiphase DENSE‐MRE images. Regional assessment of the underlying values was done using the same ROIs copied to the respective maps.

### 2.4 Brain MRE

Four healthy volunteers (male, age range 27‐48) were investigated to evaluate the in vivo performance of the multiphase DENSE‐MRE acquisition scheme. The study was approved by the local ethics committee and all subjects gave written informed consent. In these investigations, 5 slices were acquired using the same setup and acquisition protocol as in the phantom experiments except for the excitation frequency, which was reduced to 20 Hz. However, the mixing times of the 20‐Hz in vivo acquisitions still matched the mixing times of the 60‐Hz phantom experiments. Thus, for the in vivo as well as for the phantom experiments, the same sequence‐parameter settings were used for the multiphase DENSE‐MRE acquisition. Combined with the same TR of 2500 ms, the acquisition time of 2 minutes 30 seconds per motion‐encoding direction also did not change between phantom and in vivo investigations. Postprocessing was performed as described previously for the phantom experiment with only slight adoptions. Because the mixing times correspond to the ascending order of the wave sampling points (Table 1), no rearrangement of the data set was necessary. Due to the longer wavelengths that result from the lower excitation frequency, during the inversion the Butterworth filter threshold was set to 50 m^−1^ as in Ref 10, and no median filtering of the G* maps was performed. For all subjects, the mean values of the magnitude and phase angle of the complex shear modulus were assessed. Global mean |G*| and φ were measured across all slices covering gray and white matter and excluding the ventricles. The outline of the brain was defined on the DENSE‐MRE magnitude images using a threshold, and the T_2_‐weighted images were used to manually exclude the ventricles from the regions. Additionally, ROIs were manually defined on the T_2_‐weighted images in the white matter of the frontal and occipital lobes, the centrum semiovale, and in the deep gray matter (putamen) of both hemispheres. The regions were copied to the |G*| and φ maps for evaluation, and the values of both hemispheres were averaged. The ROIs were also used to assess the SNR within the raw multiphase DENSE‐MRE magnitude images. The SNR of each phase offset time step was evaluated. Separate evaluation was performed for each motion‐encoding direction and subject. The SNR was thereby calculated as the ratio of the mean to the SD of the magnitude signal intensity within the ROIs and averaged over all ROIs. The average SNR of all subjects and motion‐encoding directions was calculated for all 4 phase offset magnitude images.

## 3 RESULTS

### 3.1 Magnetic resonance elastography in phantom

**Figure 2:**
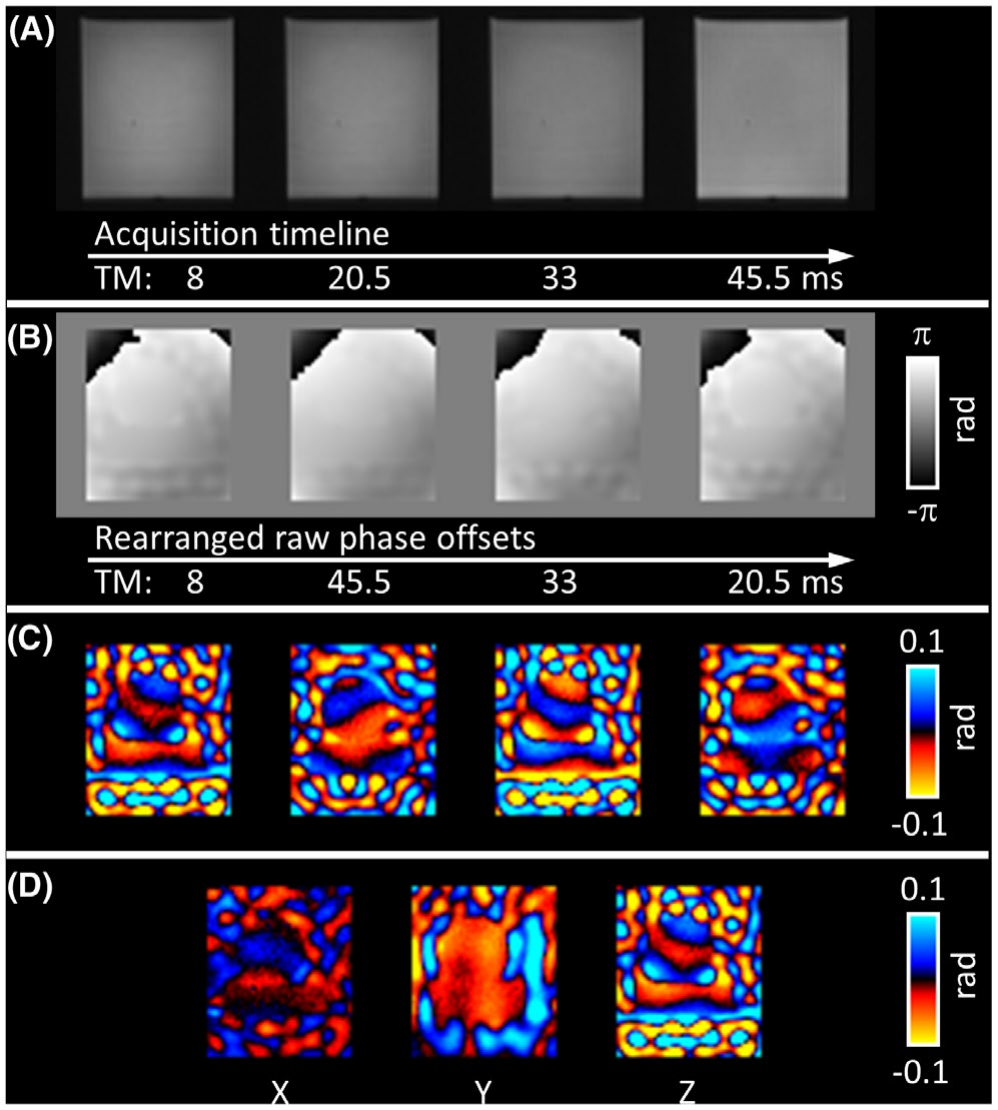
Multiphase DENSE‐MRE magnitude and phase images of the central slice of the phantom experiment. A, Artifact‐free raw magnitude images at the applied TM. B, Temporally rearranged raw phase‐offset images represent the displacement information in *z*‐direction. The time‐resolved wave information is superimposed by background phase. C, Clear wave images after background phase subtraction. D, Real parts of the first harmonics for all 3 separately acquired encoding directions. All images are presented without spatial filtering

**Figure 3:**
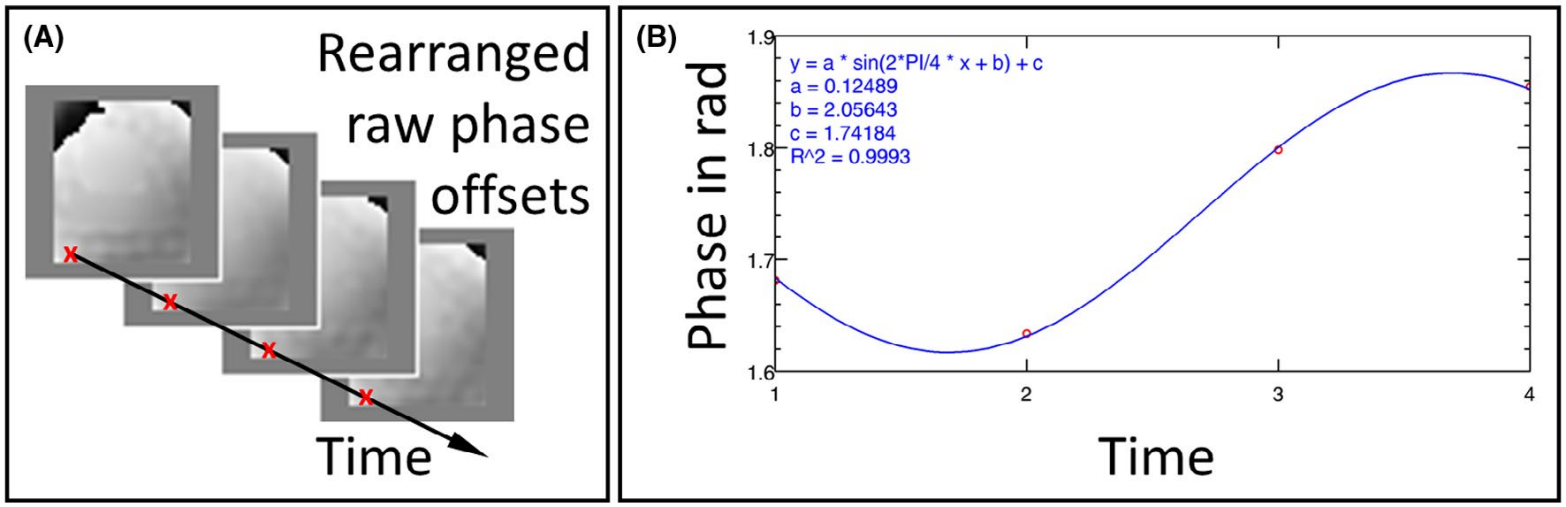
A, Rearranged raw phase‐offset images of the central slice of the multiphase DENSE‐MRE phantom experiment. B, Phase value over time of an exemplary voxel (marked in red). The fitted sine wave illustrates that the encoded phase over time is following the sinusoidal excitation motion but is shifted by a background phase

A representative set of images from the multiphase DENSE‐MRE phantom experiments is shown in Figure 2. As expected from the variable flip‐angle scheme, raw magnitude images at different phase offsets provided constant signal intensities (Figure 2A). The displacement information in the raw phase images is superimposed with background phase (Figure 2B). During postprocessing, the background phase was subtracted, which resulted in clear wave images (Figure 2C). First harmonic extraction revealed the complex wave image related to the excitation frequency. Complex wave images (real parts) for all 3 orthogonal displacement‐encoding axes are depicted in Figure 2D. All images show the displacement information before any spatial smoothing filter was applied. The displacement information in the raw phase offset image series is further demonstrated in Figure 3. The timeline plot of an exemplary voxel demonstrates that the raw phase information at the 4 phase offsets follows the sinusoidal excitation motion but is shifted by the background phase (Figure 3B).

**Figure 4:**
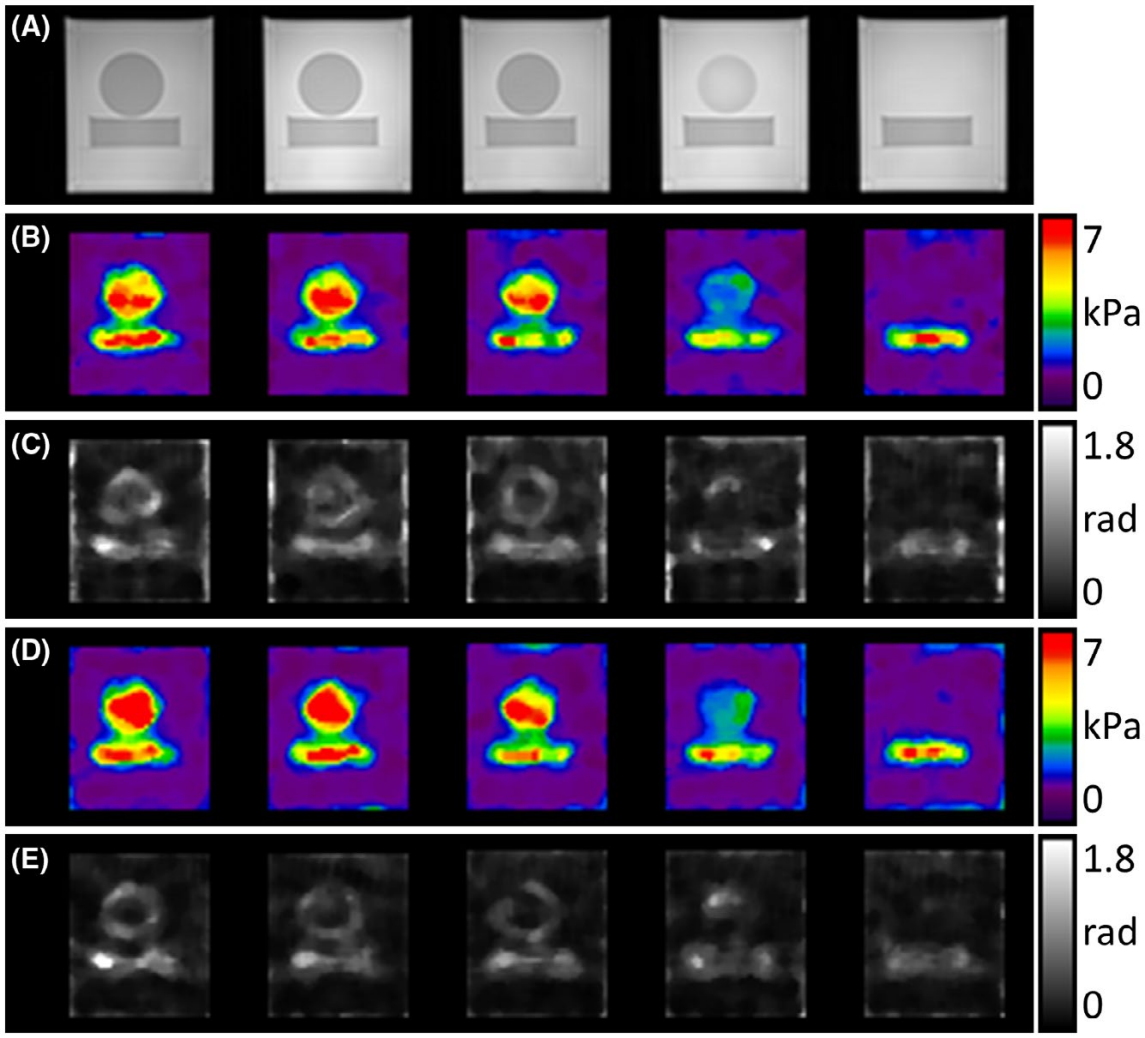
A, T_2_‐weighted images of the 5 slices in the phantom. B,C, Complex shear modulus maps obtained by multifrequency dual elasto visco (MDEV) inversion of the *z*‐displacement encoded multiphase DENSE‐MRE data set as |G*| (B) and φ (C). The stiff inclusions in the phantom can clearly be distinguished in the maps and correspond well with the phantom structures given in (A). D,E, The MDEV results of the conventional gradient‐echo (GRE)‐MRE *z*‐motion‐encoded data set as |G*| (D) and φ (E). The |G*| and φ maps match well between the proposed approach and the conventional approach

After inversion of the multiphase DENSE‐MRE data set, |G*| and φ maps were obtained and the resulting maps based on the *z*‐motion‐encoded data were analyzed. Figure 4 illustrates the corresponding T_2_‐weighted images (Figure 4A) together with the complex shear modulus maps (Figure 4B‐C). Variations of the shear modulus values were minor within areas of similar agar concentration. As confirmed by the T_2_‐weighted images, the shear modulus maps clearly resemble the structure of the phantom, with both inclusions clearly separated from each other and from the surrounding area. Even partial‐volume effects of the cylindrical inclusion in the fourth slice resulted in a slight increase in the shear modulus magnitude in the corresponding region compared with the surrounding. As expected, the higher agar concentration of 0.6% within both inclusions led to a higher mean |G*| compared with the embedding 0.3% agar bulk material, and φ was higher within the inclusions (Table 2). Figure 4 and Table 2 further summarize the results of the comparative measurements with the conventional *z*‐motion‐encoding GRE‐MRE sequence. The complex shear modulus maps generated from multiphase DENSE‐MRE acquisitions (Figure 4B,C) corresponded very well with results from the GRE‐MRE measurements (Figure 4D,E) in all 5 slices. Regional shear modulus values were also comparable for both methods (Table 2).

**Table 2:** Regional assessment of |G*| and φ in the inclusions and surrounding area of the phantom obtained by multiphase DENSE‐MRE and by conventional GRE‐MRE

	|G*| (kPa)		φ (rad)	
	Inclusions	Surrounding	Inclusions	Surrounding
Multiphase DENSE‐MRE	4.46 ± 1.48	1.25 ± 0.09	0.64 ± 0.26	0.14 ± 0.09
Conventional GRE‐MRE	4.78 ± 1.70	1.24 ± 0.08	0.52 ± 0.30	0.10 ± 0.04

Note.

Values are presented as mean ± SD.

### 3.2 Magnetic resonance elastography in the human brain

**Figure 5:**
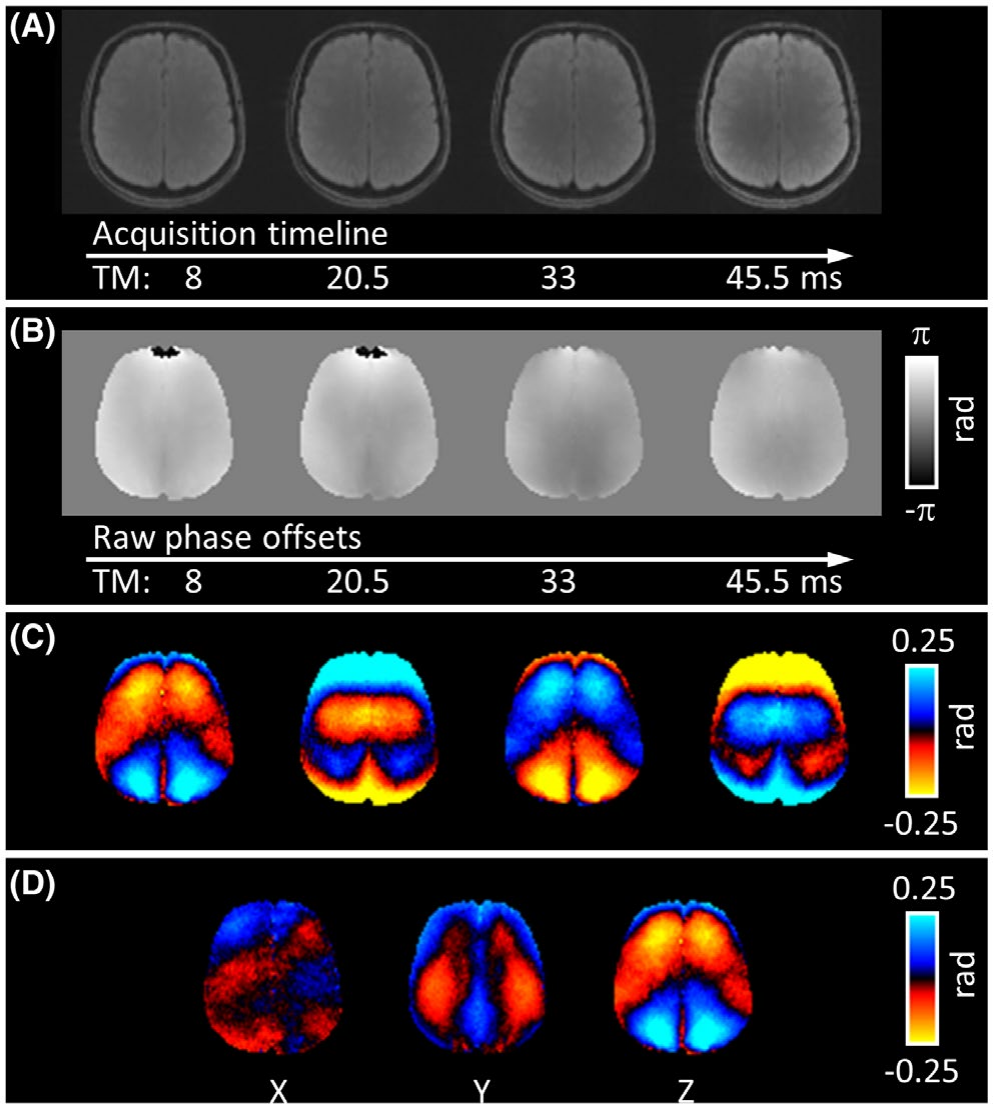
Multiphase DENSE‐MRE magnitude and phase images of 1 volunteer (1 slice). A, Artifact‐free raw magnitude images at the applied TMs. B, Raw phase‐offset images representing the displacement information in *z*‐direction. The time‐resolved wave information is superimposed by background phase. C, Clear wave images after background phase subtraction. D, Real parts of the first harmonics extracted at the 20‐Hz driving frequency for all 3 separately acquired encoding directions. All images are presented without spatial filtering

**Figure 6:**
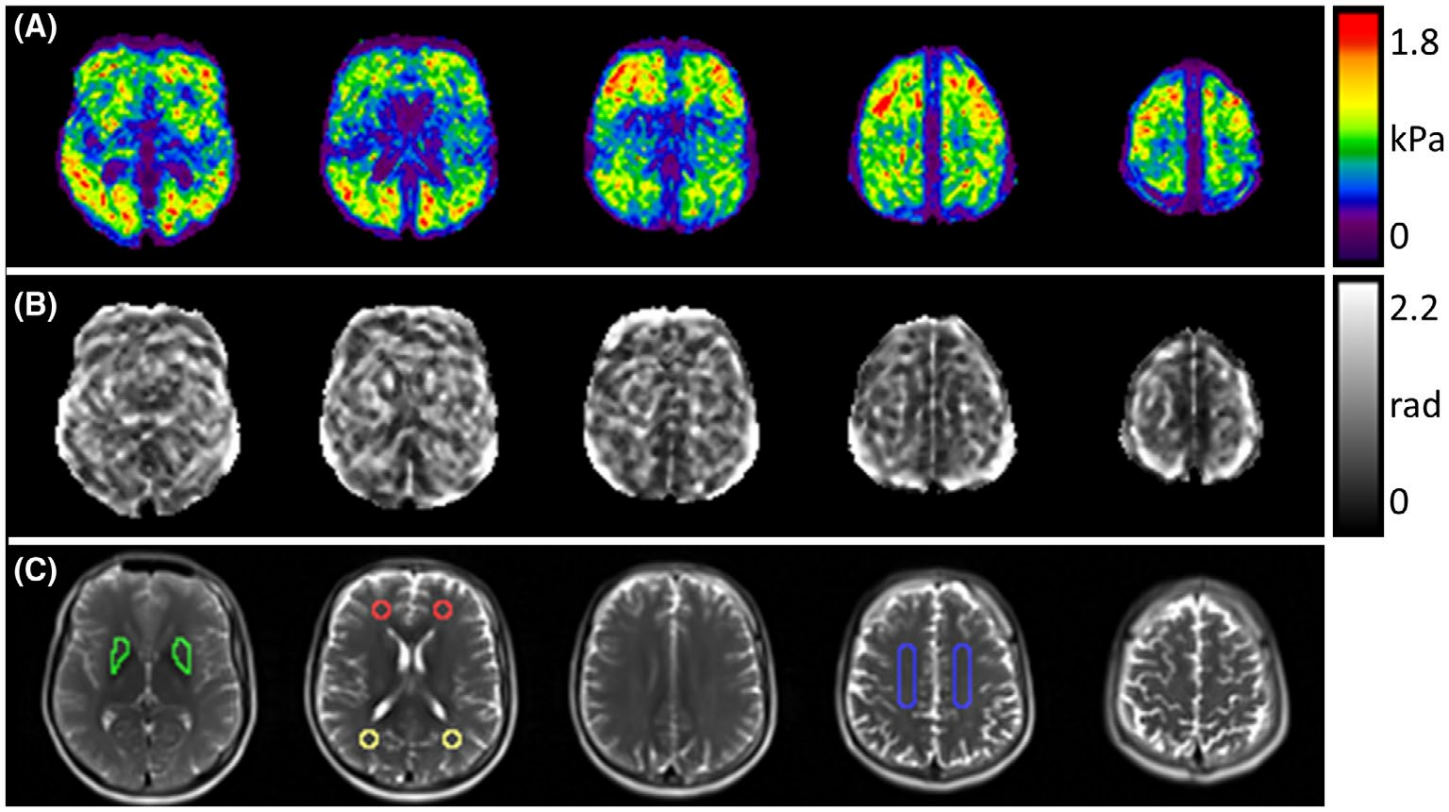
The |G*| (A) and φ (B) maps of 1 subject, together with the corresponding T_2_‐weighted images (C). The colored lines delineated in (C) describe the region of interest placing for regional |G*| and φ assessment (green, deep gray matter (putamen); red, frontal white matter; yellow, occipital white matter; blue, centrum semiovale)

One slice of the brain multiphase DENSE‐MRE data is presented in Figure 5. As in the phantom, the acquisition in the human brain provided a series of magnitude images with matching signal intensities and free of any susceptibility artifacts (Figure 5A). Rearrangement of the raw phase offset image series (Figure 5B) was skipped because the acquisition of the phase offsets was already in a chronological order (Table 1). Background phase elimination revealed the wave dynamics (Figure 5C) and first harmonic images clarified the complex wave pattern. The real parts of the complex wave images for the 3 encoding axes are illustrated in Figure 5D. All images in Figure 5 are presented without spatial smoothing. Representative shear modulus maps of 1 volunteer are presented in Figure 6A, B as magnitude and phase angle images. The defined regions of interest used for evaluation are delineated on the T_2_‐weighted images (Figure 6C). The assessed values across the volunteers are listed in Table 3 as magnitude |G*| and phase angle φ of the complex shear modulus data. The results from all 4 subjects were comparable in terms of image quality, wave patterns, and magnitude image SNR. Averaged over all subjects and motion‐encoding directions, the median SNR across the 4 phase offset points was 21.2 (ranging from 14.2‐24.9).

**Table 3:** Median (minimum–maximum) values of the magnitude and phase angle of the complex shear modulus G* across the 4 subjects assessed in different regions of the brain

Region	|G*| (kPa)	φ (rad)
Global	0.72 (0.64‐0.75)	1.03 (0.95‐1.07)
Frontal white matter	1.04 (0.96‐1.10)	1.01 (0.96‐1.26)
Occipital white matter	0.96 (0.90‐1.09)	0.95 (0.67‐1.15)
Centrum semiovale	0.83 (0.71‐1.00)	0.79 (0.71‐0.87)
Putamen	0.94 (0.64‐1.26)	1.11 (0.97‐1.19)

## 4 DISCUSSION

We propose a modified DENSE sequence with a multiphase offset readout technique for an efficient acquisition of all dynamic phase offset scans required for an MRE examination at a low excitation frequency. The multiphase DENSE‐MRE uses displacement encoding through stimulated echoes at several excitation‐frequency matched readouts to fully sample the mechanical waves during 1 sequence execution. Stimulated echo imaging with acquiring multiple k‐space lines through multiple readouts during a single TR is a well‐known concept for reducing acquisition time.^17^ The novelty in the proposed concept is the acceleration resulting from acquiring all time‐resolved phase offsets during each TR in a single sequence run. This brings along the major benefit that displacement encoding of all dynamic phase offset images is performed as short as possible in time. Consequently, influences like misalignment of k‐space lines due to patient movement or magnetic field variations over time are kept to a minimum when phase contrast images are reconstructed. This is a known concept from velocity‐encoded MRI with bipolar gradients^18^ and is therefore also favorable in MRE when the first harmonic complex wave images are extracted. In addition to the compact acquisition of all phase offsets, the application of DENSE in MRE offers several benefits. The DENSE‐MRE technique allows the use of short TEs, which is beneficial in tissues with short T_2_ and T_2_^*^ values^14^ and for preventing susceptibility artifacts. Additionally, in DENSE‐MRE, the displacement‐encoding gradients and the excitation frequency are decoupled from each other,^14^ which easily enables MRE acquisitions at low vibration frequencies. Moreover, the proposed approach allows the use of interleaved multislice imaging, which further reduces the total acquisition time for whole‐brain investigations. In this work, 5 slices were acquired. The 5 slices should demonstrate the interleaved slice acquisition capability of the sequence but not represent the maximum possible number of slices. By matching TR together with the number of slices precisely to integer multiples of the wave period time, 2 issues can be addressed. First, when also triggering the actuator consistently while avoiding delay times, a continuous harmonic excitation throughout a single sequence execution is achieved. Thus, a steady‐state condition of the wave propagation within the tissue is ensured during the acquisition of the complete phase offset image series and for all slices. Continuous application of the vibration for MRE was also pointed out to be beneficial by Dittmann et al.^10^ Second, the proper multislice timings also allow slice‐wise sampling point matching in the time‐resolved wave fields. In contrast, neglecting the slice‐wise sampling point matching at the moment of acquisition allows one to increase the number of interleaved slices. During postprocessing, however, as introduced by Dittmann et al,^10^ the sampling point differences between the slices can be matched again by shifting the phase of the complex wave images for each slice accordingly.

In the phantom experiments, the shear moduli of the inclusions and surrounding matrix corresponded well with the respective agar concentrations. Phantom components made of higher agar concentration are known to be stiffer than lower agar concentrated parts,^5^ which showed up in the |G*| maps together with raised mean |G*| values in the inclusions compared with the surrounding. Comparison of the multiphase DENSE‐MRE with the conventional GRE‐MRE showed the validity of the proposed approach. Both the magnitude and phase angle of the complex modulus were visually similar in all 5 slices. Furthermore, the assessed mean values of |G*| and φ corresponded well between the 2 acquisition techniques.

The feasibility of multiphase DENSE‐MRE for brain MRE was further demonstrated in healthy volunteers. Even though the excitation frequency was changed between the 60‐Hz phantom measurements and the 20‐Hz in vivo investigations, the sequence parameters could be kept unchanged. The low‐frequency experiments with 20 Hz yielded a median global |G*| of 0.72 kPa. Dittmann et al^10^ also assessed |G*| in healthy human brain in a frequency range of 10 Hz to 20 Hz. They investigated 8 healthy volunteers using a motion‐encoding EPI‐based MRE sequence and found a mean |G*| of 0.62 ± 0.08 kPa (mean ± SD), which corresponds well to the global values presented here. Our results of the regional |G*| of white matter and gray matter areas are higher than the global value, which suggests that partial‐volume effects with cerebrospinal fluid may reduce global |G*|.

In multiphase DENSE‐MRE, the acquisition of the sampling points is not restricted to a chronological order. Optimized ordering during acquisition and rearrangement of the phase offset images in postprocessing offers some advantages. It helps to keep the intervals between the readouts short and increases overall efficiency. This becomes especially relevant at higher excitation frequencies like in the 60‐Hz phantom experiment. Shorter intervals between readouts allow a higher number of slices for a given TR. At the low frequency of 20 Hz, the 4 readouts used in this work were short enough and allowed a chronological order; thus, ascending phase‐offset acquisition without rearrangement was applied. Nevertheless, the concept of multiphase DENSE‐MRE is not restricted to only 4 readouts. Future developments may benefit from a higher number of phase offsets and a corresponding sampling reordering even at low excitation frequencies. In this work we performed the phantom and in vivo examinations with the same imaging sequence parameters to demonstrate the capability of the sequence to acquire wave images at different excitation frequencies without the need of adapting sequence parameters. Basically, this concept is not restricted to the applied driving frequencies, but can also be adjusted to any frequency or combination of frequencies by changing the set of mixing times.

The image series acquired with the multiphase DENSE‐MRE approach contains a constant offset along the time axis of the data set. In this work we subtracted this constant temporal mean to visualize the waves before the first harmonic Fourier extraction. The first harmonic extraction as well as the temporal mean value subtraction, however, bring along a limitation, as both of these techniques only work if there are no phase wraps in the temporal signal.

Another limitation of the proposed approach arises from the type of signal echo used. In general, the use of stimulated echoes for image acquisition reduces the longitudinal magnetization, and therefore the maximum signal, by a factor of 2 compared with spin‐echo imaging.^17^ In contrast, there is less signal loss from transversal relaxation due to short TEs achievable in DENSE‐MRE. However, the short TE in this work also required short motion‐encoding gradients that consequently reduced the motion‐encoding sensitivity for the wave motion. In contrast, prolonging the motion‐encoding gradients would result in a higher motion‐encoding sensitivity, and a tradeoff between short TE and motion‐encoding sensitivity has to be found.

With the proposed multiphase approach, the total acquisition time can be reduced. As all phase offsets are acquired at once, the total acquisition time shortens by the factor of the number of phase offsets compared with repeated single phase‐offset acquisitions. Hence, the acceleration in this work was by a factor of 4. Higher acceleration factors come along with lower flip angles, resulting in lower signal intensities for each readout block. Consequently, the SNR reduces with higher acceleration factors. Additionally, variations of T_1_ are then more difficult to address with the flip angle variation scheme. The T_1_ relaxation also forces the TR to be long in the proposed sequence compared with conventional GRE‐MRE. Thus, the scan time efficiency in multiphase DENSE‐MRE increases with higher numbers of phase offsets and acquired slices. Current EPI‐based MRE sequences with fractional motion encoding can achieve shorter acquisition times (for example, Dittmann et al^10^ scanned 1 minute per frequency at 2 × 2 × 2 mm resolution) than the presented approach. However, DENSE provides a fully refocused echo that is free of EPI distortions and susceptibility artifacts. Future multiphase DENSE‐MRE studies could benefit from fully exploiting the number of interleaved slice acquisitions without a change in scan time. Additionally, the advantages of the proposed approach could likely be even more relevant when investigating organs with short T_2_ and T_2_^*^ values. Furthermore, future studies should explore whether combining the multiphase DENSE‐MRE approach with EPI readouts may allow a further reduction of the acquisition time, and whether a conventional motion‐encoding EPI‐based MRE sequence was still more efficient than the proposed approach.

## 5 CONCLUSIONS

In this study we showed the feasibility of acquiring all MRE relevant phase‐offset scans using a DENSE approach with a multiphase acquisition. Our results demonstrate that the sequence is well‐suited for low‐driving frequencies and that the images do not suffer from susceptibility artifacts. With the multiphase expansion, the total acquisition time can be significantly shortened, which allows efficient multislice MRE of the brain in a clinical setting.
